# Antitoxic Effects of Curcumin against Obesity-Induced Multi-Organs' Biochemical and Histopathological Abnormalities in an Animal Model

**DOI:** 10.1155/2022/9707278

**Published:** 2022-10-06

**Authors:** Mohammed H. Hassan, Eatemad A. Awadalla, Abd El-Kader M. Abd El-Kader, Esraa A. Seifeldin, Marwa Ahmed Mahmoud, Abdel Rahim Mahmoud Muddathir, Ahmed Abdelsadik

**Affiliations:** ^1^Department of Medical Biochemistry, Faculty of Medicine, South Valley University, Qena 83523, Egypt; ^2^Department of Zoology, Faculty of Science, Aswan University, Aswan 81528, Egypt; ^3^Department of Medical Physiology, Faculty of Medicine, Sohag University, Sohag, Egypt; ^4^Department of Hematology and Blood Transfusion, Faculty of Medical Laboratory Science, Alzaeim Alazhari University, Khartoum, Sudan

## Abstract

**Background:**

Obesity is a significant public health problem that is characterized by an increase in oxidative stress and enhanced inflammatory responses associated with immune cell invasion of adipose tissues. This study assessed several biochemical abnormalities, apoptosis, oxidative stress status, and associated histological changes in the liver, duodenum, and heart brought on by high-fat diet-induced obesity in rats. It also assessed the mechanistic benefits of curcumin in reversing these inflammatory, metabolic, and histological impairments.

**Methods:**

Rats were assigned into three groups each including ten rats: the control group (CD), the high-fat diet group (HFD), and the high-fat diet + curcumin (HFDC) group. Serum glucose, insulin, and triglycerides (TAGs) were observed. In addition, apoptosis (indicated by hepatic DNA fragmentation) and oxidative stress status (indicated by hepatic MPO, GSH, and SOD) were assessed. Histopathological examinations included the GIT (liver and duodenum) and heart in addition to quantitative real-time polymerase chain reaction (qRT-PCR) assays of the adipose tissue genetic expressions for inflammatory signaling pathways (TLR4, IL-6, and TNF-*α*).

**Results:**

The overall findings showed that the HFD group exhibited significantly higher levels of glucose, TAGs, and insulin than the control group (*P* < 0.01). The histological abnormalities of the studied organs in the HFD group were paralleled by these biochemical abnormalities, which were strongly associated with increased apoptosis, increased oxidative stress, and increased expression of the inflammatory signaling markers. There were significant improvements in the HFDC group in terms of biochemical, inflammatory, and histological investigations.

**Conclusions:**

This study's findings concluded that obesity is significantly associated with biochemical and microscopic alterations in many organs. Curcumin exerted potent antitoxic, antioxidant, tissue-protective, and antiobesity effects. Curcumin is recommended to be added to various dietary regimens to prevent or delay the organs' dysfunction among obese people.

## 1. Introduction

Obesity is a metabolic condition that is chronic and associated with a low-grade inflammatory response. Not only in the West but also in many developing countries, obesity is a major public health issue. Obesity is a significant risk factor for cardiovascular diseases, such as hypertension, metabolic disease, and diabetes mellitus [1, 2].

Because of its low satiety properties and high caloric density, a high-fat diet (HFD) has been linked in epidemiological studies to the development of obesity by encouraging food overconsumption and weight gain. Furthermore, HFD alters the composition of gut microbes in a way that increases the energy extraction of indigestible dietary components, increasing the efficiency of food utilization [3].

Free fatty acids (FFAs) and hormones called adipokines are secreted by adipose tissues, which may be a crucial factor in the emergence of non-alcoholic fatty liver diseases (NAFLD). It is reported that toxic FFAs can cause c-Jun N-terminal kinase (JNK) to activate the intrinsic apoptotic pathway in hepatocytes. The proapoptotic protein Bim is activated by JNK, which also activates Bax and causes lipoapoptosis. Reduced adiponectin levels could create a proinflammatory environment, making people more susceptible to lipotoxicity, which encourages the development of non-alcoholic steatohepatitis (NASH) and even advanced hepatic fibrosis from simple steatosis [4].

According to previous publications, macrophages invade adipose tissues more as the level of obesity rises. An essential cell type in mediating the inflammation brought on by obesity in the adipose tissue is the macrophage. These macrophages may be the main source of cytokines that promote inflammation, e.g., interleukin-1 (IL-1), interleukin-6 (IL-6), and tumor necrosis factor-*α* (TNF-*α*). According to recent research, obesity is characterized by an increase in oxidative stress and amplified inflammatory reactions that occur along with immune cells infiltrating adipocytes [5, 6]. The antioxidant system of the living system possesses its own antioxidant defense that includes major antioxidant enzymes such as superoxide dismutase (SOD), catalase (CAT), glutathione peroxidase (GPx), and reduced glutathione (GSH) [7, 8]. Myeloperoxidase (MPO) is currently thought of as a novel inflammatory biomarker in acute coronary syndrome and ischemic heart diseases [9]. To kill pathogens, MPO produces a variety of oxidants, but it also damages the host's tissues [10].

A class of conserved intracellular and cell surface proteins called toll-like receptors (TLR) identify different pathogen-associated molecular patterns (PAMPs) and trigger innate immune reactions. The synchronization of immune response and nutrient-sensing pathways is made possible by these molecular sites. Several TLR proteins exist, but TLR2 and TLR4 are thought to be crucial metabolic inflammatory regulators throughout the emergence of obesity and associated co-morbidities [11]. It has been documented and proposed that obesity-related DNA damage has a role in the development of obesity-related diseases [12]. Obesity-related DNA damage appears to be repairable, and changes in diet and exercise routines can have an impact on genomic stability [13].

Turmeric (*Curcuma longa*) has more than 100 components that have been identified. Turmeric contains additional coloring compounds known as curcuminoids in addition to its volatile oil, which is the root's major component and contains turmerone. Turmeric contains natural antioxidants known as curcuminoids, including curcumin dimethoxy curcumin, 5′-methoxycurcumin, and dihydro curcumin [14, 15]. The most significant phenolic ingredient in turmeric is called curcumin, and it is yellow in color and is a natural phenolic antioxidant [16]. Due to its biological and pharmacological characteristics, which include antioxidant and anti-inflammatory features, curcumin has received a lot of interest in various research studies, including human or experimental animal models in various diseases, including obesity [17].

In light of the previous research efforts, it is crucial to assess the different biochemical abnormalities caused by high-fatdiet-induced obesity in rats, including glucose homeostasis, lipid profile, apoptosis (via assessment of hepatic DNA fragmentation), and oxidative stress status (via assessment of hepatic MPO, GSH, and SOD), as well as any possible associated histopathological changes in the GIT (liver and duodenum) and heart. Additionally, genetic analyses of the related inflammatory signaling pathways (via assessments of adipose tissue expressions of TLR4, IL-6, and TNF-*α*) were performed to explore the potential mechanistic benefits of curcumin in reversing these metabolic abnormalities.

## 2. Materials and Methods

### 2.1. Experimental Design

**Figure 1:**
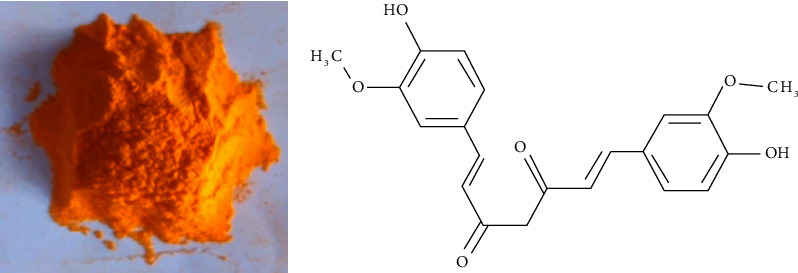
Curcumin. (a): Curcumin powder; (b): chemical structure of curcumin.

Thirty male albino rats were 50–60 grams when the experiment started. Before the study began, rats were procured from the Qena Breeding Center and given a week of acclimatization. Rats were divided into three groups at random. At a temperature of 25°C and a 12-hour cycle of dark and light, all groups were maintained in the same habitat. All experimental groups received the same feeding schedules and water supplements. Animals were kept in normal air-conditioned conditions in well-ventilated cages with sawdust bedding. According to the manual for the care and use of laboratory animals, the Aswan University Animal Ethical Committee in Egypt approved the treatment of the animals and the experimental techniques. The ethical approval code for the present study is ASW-Sci-ZOO-15-6-015 and the date of approval was January, 2020. Curcumin powder with the CAS number 458-37-7 was bought from Merck for use in this experiment (Figures 1(a) and 1(b)). The nutrition of the experimental animals include 50.17 grams of crude carbohydrate, 30.2 grams of crude protein, 6.4 grams of crude fat, 4.3 grams of crude fiber, 5.8 grams of mineral mixture, and 1.6 g of vitamin mixture/100 g diet, which made up the mash. To harmonize the dietary amounts utilized in various types of groups, we used 20 grams of beef grease in the high-fat diet. The rats were randomly divided into three groups, 10 rats each. The groups were assigned to a food type as follow:

- Control diet group (CD): This group fed 40 grams of mash.
- High-fat diet group (HFD): This group fed on 20 grams mash + 20 g beef grease (50% fat, wt./wt.) as was previously reported [18].
- High fat diet + curcumin (HFDC): This group fed on a high fat diet + 100 mg/kg body weight curcumin as was previously reported [19]. Each rat received 5-6 mg of curcumin on average.

As stated in a previously published work [20], the rats were treated daily for 8 weeks.

### 2.2. Body Weight Measurement

Rats having an equal initial body weight were assigned to the experimental diet for 56 days. Two times a week during the trial, body weight was recorded (56 days). All groups were then given unrestricted access to their respective diets [21, 22].

### 2.3. Collection and Preparation of Blood and Tissue Samples

Rats from various groups were decapitated and then scarified twenty-four hours after receiving the final dose of treatment. Blood samples were taken from the retro-orbital veins during scarification and placed in plain tubes. These tubes were then centrifuged at 4000 rpm for 10 minutes to separate the serum. The separated sera were then divided into aliquots using 1 ml cryotubes and stored at −80°C until used in subsequent serum biochemical analyses (serum glucose, triglycerides, and insulin assays).

From each group, samples of adipose tissues, liver, duodenum, and heart were taken and cleaned in sterile saline. Three portions of each liver sample were separated. For histological analysis, the first portion of the liver, the duodenum, and the heart were preserved in 10% neutral phosphate-buffered formalin (pH 7.0).

The supernatant was used to measure the activities of GSH, SOD, and MPO after homogenizing the second portion of liver tissues using phosphate buffer (pH 7.4), a tissue homogenizer (Glas-Col USA), and centrifugation at 4000 rpm for 30 min at 4°C.

Additionally, the final third portion of the hepatic tissue samples was used to measure DNA fragmentation as a potential marker for apoptosis using spectrophotometry.

For molecular investigations of TLR4, IL-6, and TNF-*α* tests by quantitative real-time PCR (qRT-PCR), pieces of adipose tissues were preserved in liquid nitrogen and kept at −80°C.

### 2.4. Biochemical Assays

(A) Serum insulin assays were performed by a microplate ELISA reader (EMR-500, USA), using commercially available ELISA assay kits (supplied by Chongqing Biospes Co., Ltd, China with catalog number: BEK1243). Serum glucose assays were performed by the o-toluidine method as was previously reported [23] and were supplied by (Sigma-Aldrich, Germany) with CAS number 119-93-7. Serum triglyceride concentrations were estimated by the GPO-PAP- enzymatic colorimetric method as was previously reported [24], supplied by (Spectrum Diagnostics, Egypt, catalog number 314-001).
(B) determination of total protein concentration in liver tissue homogenates: Total protein content in liver tissue was estimated by the Lowery technique and results were expressed as mg/total weight hepatic tissues [25] and were used for biochemical assessment of hepatic MPO, GSH, and SOD using colorimetric kinetic methods as follows
  1. Assays of hepatic MPO activity in the liver was measured by a modified procedure from earlier described methods [26, 27]. 400 mg of liver tissue samples were homogenized in 0.5 ml of 50 mM potassium phosphate buffer with a pH of 6.0 and centrifuged at 12,000 × *g* for 20 min. at 4°C. The pellets were then suspended in 2 ml of 50 mMPB containing 0.5% hexadecyltrimethylammonium bromide (HETAB) from Sigma-Aldrich (PRD.NO. H5882). After three freeze and thaw cycles, with a vortex between cycles, the samples were centrifuged at 12,000*g* for 20 min. Aliquots (0.3 ml) were added to 2.3 ml of reaction mixture containing 50 mM O-dianisidine (Sigma, D9143) and 20 mMH_2_O_2_ solutions. A yellow compound was produced which has a maximum absorption peak of 460 nm. One unit of enzyme activity was defined as the amount of MPO present that caused a change in absorbance and measured at 460 nm for 3 min. MPO activity was expressed as U/mg tissue.
  2. Hepatic GSH concentration assays were based on the reduction of 5.5′ Dithiobis (2-Nitrobenzoic acid DTNB) with glutathione (GSH) to produce a yellow compound [28].
  3. Hepatic SOD activity was measured in tissues homogenate based on its inhibitory action on the phenazine methosulphate (PMS) that mediated reduction of the Nitroblue tetrazolium (NBT) dye [29].

### 2.5. Molecular Assays

#### 2.5.1. Quantitative Real-Time PCR (qRT-PCR)

As was previously reported, tissue total RNA was extracted from the adipose tissue (AT) using phenol/chloroform [30]. RNA was dissolved in water treated with dimethyl pyrocarbonate (DEPC-H_2_O). Using a nanodrop/spectrophotometer, the RNA content and sample quality were assessed. A ratio of roughly 1.8 and 2.0 for RNA and DNA, respectively, was taken into consideration. RNA that met the requirements for quantity and quality was kept at −80°C for future assays.

First-strand DNA was created using oligo-dT primers and Superscript RT-II reverse transcriptase (Invitrogen). Utilizing the Power SYBR Green PCR Master Mix, PCR was carried out (Applied Biosystems). On the Applied Biosystems StepOnePlus real-time PCR system, TLR4, IL-6, and TNF-*α* were examined. Primer information was provided (Table 1), and results were standardized to the housekeeping gene GAPDH as a reference [31, 32]. Calculations for the relative gene expression profile analysis were made using the ΔΔCT method equation [33], according to the following formula: ΔCT (gene) = CT (gene, sample)−CT (gene, control). ΔCT (reference) = CT (reference, sample)−CT (reference, control). ΔΔ CT = ΔCT (gene)−ΔCT (reference). Due to exponential nature of PCR, “fold change” was calculated as 2^−ΔΔCT^ [31].

#### 2.5.2. DNA Fragmentation Assays

DNA fragmentation (as a defiant marker for apoptosis in the hepatocytes) was assayed as was previously reported [34, 35], with minor modifications. 100 mg of tissue samples were dissected and minced finely with a razor blade/scalpel. The minced tissue was transferred to a microfuge containing 0.5 ml of hypotonic lysing buffer (10 mM Tris-HCl, 1 mM EDTA, pH 7.4, 0.2% Triton X-100) followed by mixing well for 5 minutes via vortex at low power. The lysates were immediately centrifuged at 12000*g* for 15 minutes to separate intact from fragmented chromatin. Supernatants and pellets were precipitated in separate microfuge tubes for 16 to 48 hours at −20°C in 50% isopropanol and 0.5 M/L NaCl. The precipitates were pelleted by centrifugation at 12000*g* for 10 minutes, air dried, suspended in TE buffer (10 mM Tris-HCl, 1 mM EDTA, pH 7.4) and heated at 55°C for 10 minutes. In this DNA fragmentation assay, intact chromatin and oligonucleosomal fragments were separated by centrifugation. DNA fragmentation was expressed spectrophotometrically at 260 nm using Thermo-Nanodrop using formula (1):

(1) $$\begin{array}{c} \%\text{DNA fragmentation}=\frac{\text{Optical density}\left(\text{O.D}\right)\text{ of the supernatant}}{\text{O.D.Supernatant}+\text{O.D.Pellet}}\times 100. \end{array}$$

### 2.6. Histological Examination of the Liver, Duodenum, and Heart Tissues

Liver, duodenum, and heart specimens were dehydrated gradually in ethyl alcohol (50–99%), cleaned in methyl benzoate, and embedded in molten paraffin wax at 58–62°C for microscopic preparations. For microscopic examination, tissue slices with a 5 *μ*m thickness were produced and stained with hematoxylin and eosin as reported previously [36]. Under a high-power light microscope, the sections were examined to detect histological and histopathological changes (Olympus BX43F, Tokyo, 163-0914, Japan). Image analysis was carried out using a personal computer, a camera, software (Olympus DP74 Tokyo 163-0914, Japan) and an optical microscope.

### 2.7. Statistical Analysis

The obtained data were expressed as means ± SEM. Differences between means were tested by one-way analysis of variance (ANOVA) followed by the Student–Newman–Keuls *T*-test using Minitab 12 software so that the data obtained can be compared and statistically evaluated. Statistical significance was considered when *P* < 0.05.

## 3. Results

**Figure 2:**
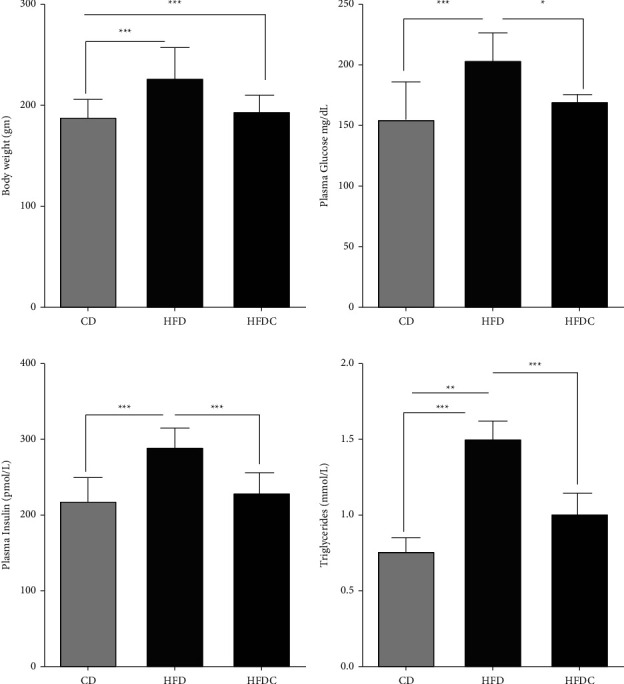
Mean values of (a) body weight (gm), (b) serum glucose (mg/dL), (c) insulin (pmol/L), and (d) triglycerides levels (mmol/L). Values are expressed as mean ± SEM. The sample size was *n* = 10 animal per group. CD, control diet; HFD, high-fat diet; HFDC, high-fat diet + curcumin. ^*∗*^indicates *P* < 0.05, ^*∗∗*^indicates *P* < 0.01 and ^*∗∗∗*^indicates *P* < 0.001.

The data regarding body weight were recorded as previously published [36]. Briefly, the mean ± SEM of body weights of the HFD group (227.22 gm ± 25.88) was significantly increased compared with the CD group (190.88 gm ± 22.88), while it significantly decreased in the HFDC group (199.25 ± 14.33) compared with the HFD group, *p* < 0.05 for all (Figure 2(a)).

### 3.1. Serum Glucose, Insulin, and Triglycerides in Various Study Groups

There were significantly (*P* < 0.01) increased serum glucose, insulin, and triglycerides in the HFD group as compared with the CD group. Coadministration of curcumin in the HFDC group exhibited significantly (*P* < 0.05) decreased serum glucose, insulin, and triglycerides when compared with the HFD group (Figures 2(b)–2(d)).

### 3.2. Hepatic DNA Fragmentations, MPO, GSH, and SOD Activities among the Included Study Groups

**Figure 3:**
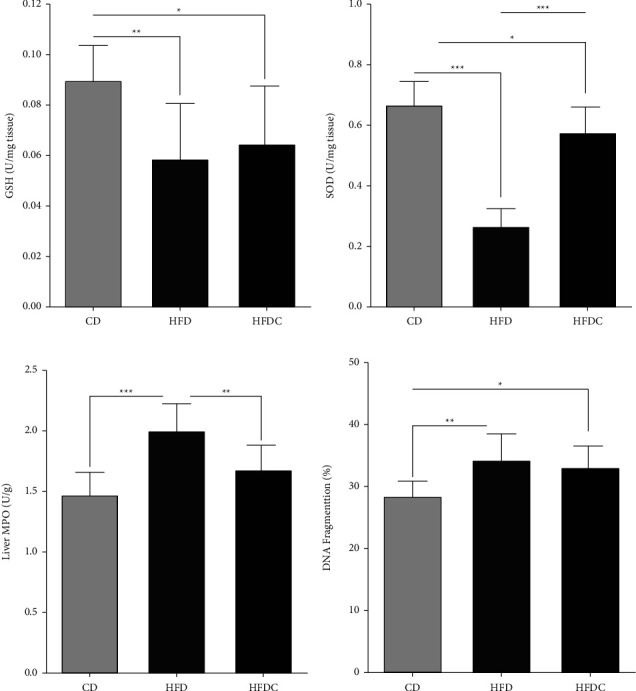
Hepatic profile of (a) glutathione reduced (GSH), (b) superoxide dismutase (SOD), (c) myeloperoxidase activity (MPO), and (d) DNA fragmentation percentage of control and treated rats. Each group consists of 10 rats. Values are represented as mean ± SEM. Bars without a common letter is not differ while ^*∗*^indicates *P* < 0.05, ^*∗∗*^indicates *P* < 0.01 and ^*∗∗∗*^indicates *P* < 0.001.

Fragmentation of DNA was significantly increased (*P* < 0.01) and MPO activity with significantly decreased GSH and SOD levels in the liver homogenates of the HFD group compared to the CD group. However, curcumin had caused a significant decrease in DNA fragmentation and MPO activity that was associated with significantly higher levels of GSH and SOD in the HFDC group when compared with the HFD group (Figures 3(a)–3(d)).

### 3.3. Adipose Tissue Genetic Expressions of TLR4, IL-6, and TNF-*α* of the Control and Different Treated Groups

**Figure 4:**
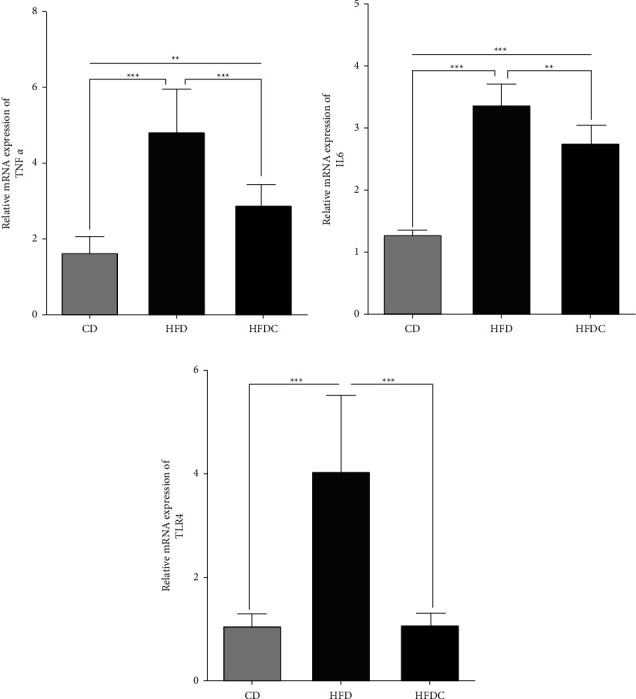
Alterations of adipose tissue inflammatory markers between control fed with control diet (CD) and those fed with high-fat diet without curcumin (HFD) or with curcumin (HFDC). The relative mRNA expression was demonstrated by reverse transcription-quantitative polymerase chain reaction. (a) tumor necrosis factor-alpha (TNF-*α*), (b) interleukin-6 (IL-6), and (c) toll like receptor-4 (TLR4). Each experiment was repeated three times. Values are given as the mean ± the standard error. ^*∗*^*P* < 0.05, ^*∗∗*^*P* < 0.01 and ^*∗∗∗*^*P* < 0.001.

Levels of TLR-4, IL-6, and TNF-*α* gene expressions of adipose tissues were significantly (*P* < 0.01) higher in the HFD group than in the CD group. In comparison with the HFD-fed rats, the HFDC group showed significantly (*P* < 0.01) lower levels of the previously mentioned three genes' expressions in adipose tissues (Figures 4(a)–4(c)).

### 3.4. Histopathological Findings of Liver, Duodenum, and Heart Tissues from Various Study Groups

#### 3.4.1. Histopathological Findings of the Liver

**Figure 5:**
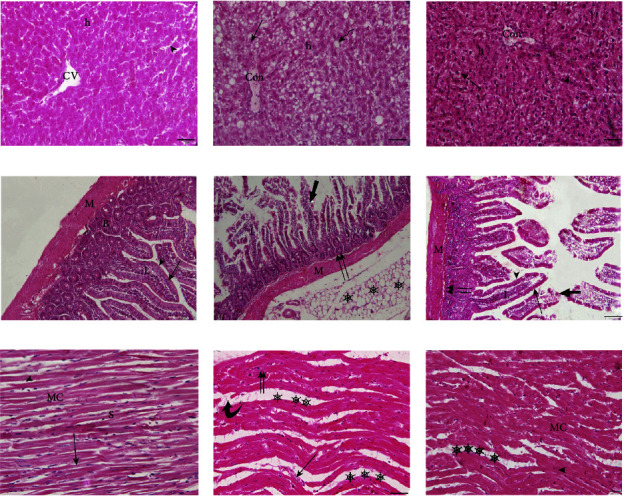
Histopathological findings of liver, duodenum and heart of various study groups. (a–c): Photomicrographs of male albino rat's liver stained with haematoxylin and eosin (HE). (×200 = 100 *μ*m). (a): Liver photomicrograph of the CD group. (b): Liver photomicrograph of the HFD group. (c): Liver photomicrograph of the HFDC group. Central vein (CV), hepatocytes (h), blood sinusoids (arrowhead), congested central vein (Con), macrovesicular steatosis (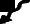), and microvesicular steatosis (thin arrow). (d–f): Photomicrographs of male albino rat's duodenum stained with haematoxylin and eosin (HE). (×200 = 100 *μ*m). (d): Duodenal photomicrograph of the CD group. (e): Duodenal photomicrograph of the HFD group. (f): Duodenal photomicrograph of the HFDC group. Musculosa (M), Brunner's glands (B), Lamina propria (L), simple columnar epithelium of villus (thin arrow), Goblet cells (arrow head), epithelial erosion (thick arrow), inflammatory infiltration (I), Necrosis (double arrow) of the inner circular muscle fibers of musculosa, Massive amounts of adipose tissues (stars). (g–i): Photomicrographs of male albino rat's heart stained with haematoxylin and eosin (HE). (×400 = 50 *μ*m). (a): Heart photomicrograph of the CD group. (b): Heart photomicrograph of the HFD group. (c): Heart photomicrograph of the HFDC group. Muscle fibers (MC), the intercellular spaces (S), vesicular nuclei (arrow), intercalated discs (I) (arrow head), damage of cardiac muscle fibers (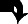), inflammatory cells (double arrows), separation of cardiac muscle fibers (stars), and extravasated red blood cells (thin arrow).

Microscopic examinations of the CD group liver showed normal hepatic architecture including the normal arrangement of the cords or plates of hepatocytes, central veins with the endothelial lining, and hepatic sinusoids which were extending in between the hepatic cords (Figure 5(a)). Liver sections of the HFD group rats showed disarrangement in the architecture of hepatic cords with widespread intracellular vacuolization of hepatocytes and congested central veins with irregular outlines. Macrovesicular steatosis and microvesicular steatosis were also detected (Figure 5(b)). Cotreatment with curcumin showed moderately congested central vein and a few hepatocytes with microvesicular steatosis (Figure 5(c)).

#### 3.4.2. Histopathological Findings of the Duodenum

Microscopic examination of the duodenal sections of CD group (Figure 5(d)) revealed that the duodenum has the normal structure and is similar to all the other hollow organs of the gastrointestinal tract: mucosa, submucosa, muscularis, and serosa. Duodenal sections of the HF group (Figure 5(e)) revealed severe distortion of duodenal histoarchitecture; villi appeared with epithelial erosion and lamina propria infiltrated with inflammatory cells as well as degeneration of Brunner's glands and necrosis in the inner circular muscle fibers of musculosa. Massive amounts of adipose tissue around the duodenum were also observed. Co-treatment with curcumin showed significant improvements in the duodenal tissues of HFDC group (Figure 5(f)). The duodenal histoarchitecture of the HFDC group showed a good appearance versus to that of the duodenal HFD group, and the four layers of the duodenum displayed normal appearance to a large extent. However, degeneration and desquamation at the tips of some villi and necrosis in the inner circular muscle fibers of musculosa were discerned.

#### 3.4.3. Histopathological Findings of the Heart

Microscopic investigations of the heart of the CD group (Figure 5(g)) revealed a normal myocardial structure; branching and anastomosing muscle fibers with centrally located oval vesicular nuclei. Cardiac muscle cells appeared adherent to each other by intercalated discs that appeared as thin, typically dark-staining lines dividing adjacent cardiac muscle cells. Inspection of the (Figure 5(h)) revealed that histological examination of the HFD rat's myocardial tissue showed abnormality compared to the CD group. Rats fed on HFD showed a widespread myocardial structure and necrosis of cardiac muscle fibers. Also, extravasated red blood cells in between the muscle fibers and little inflammatory cellular infiltration were detected. Microscopical examination of the myocardial tissue sections of the HFDC group showed a cardioprotective effect of curcumin supplementation (Figure 5(i)). The same figure revealed near the normal architecture of myocardial muscle fibers with centrally located oval vesicular nuclei and intercalated discs. However, a few necrosis and separations in muscle fibers without inflammatory cells were detected.

## 4. Discussion

According to the World Health Organization, more than 1.6 billion people (aged 15 and over) are overweight. By 2030, 51% of the world's population is expected to be obese [37, 38]. To highlight the antioxidant and anti-inflammatory effects of curcumin against HFD-induced obesity and obesity-related multiorgan dysfunction using a rat model, some biochemical and immunological parameters along with some histological examinations of the liver, duodenum, and heart were assessed.

The results of the present study showed significantly higher serum levels of TAGs in the HFD group in comparison to the CD group. The increasing serum levels of TAGs observed in the present study could be attributed to the fact that in obesity, adipose tissue dysfunction will eventually lead to abnormalities in lipid metabolism, such as hypertriglyceridemia. Also, the primary dyslipidemia related to obesity is characterized by increased triglycerides, as previously reported by Abdelsadek [39]. The serum triglyceride levels in the HFDC group showed significantly decreased levels versus those of the HFD group. Our results were in agreement with previous studies [40–42] whose studies confirmed a significant effect of curcumin in reducing serum TG concentrations, suggesting the hypotriglyceridemic activity of curcumin. Also, Seo et al. [43] emphasized that curcumin significantly lowered the hepatic activities of fatty acid synthase, beta-oxidation, plasma FFA, cholesterol, and triglyceride concentrations. Similar conclusions were reported by Zhou et al. [44], who confirmed that the hypolipidemic effect of curcumin may depend on a balance between increased lipid mobilization from adipose tissue and increased lipid uptake and excretion by other tissues.

The present study showed significantly higher serum insulin and glucose levels in the HFD group compared to the control group. The present results coincide with several researchers who confirmed that oxidative stress and inflammation are the major components responsible for the pathogenesis of insulin resistance (IR) [45–47].

The present hyperinsulinemia could be attributed to the fact that insulin primarily increases the cellular uptake of glucose. During the development of IR, hepatocytes, adipocytes, and myocytes do not respond well to insulin. The *β*-cells of pancreatic islets increase insulin release sufficiently to overcome the reduced efficiency of insulin action to maintain normal glucose tolerance. Thus, people with IR also have hyperinsulinemia. As a result of IR, cellular uptake of glucose does not occur and the blood glucose level is elevated. The hyperglycemia displayed in the present study was in agreement with previous research studies [48].

Interestingly, coadministration of curcumin with a high-fat diet group exhibited significantly decreased serum insulin and glucose levels when compared with the HFD group. In parallel with our results, Hartogh et al. [49] reported that by lowering the harmful effects of liver fat and increasing cell sensitivity to insulin, curcuminoid can lower blood glucose levels in obese rats. The present results could be attributed to the ability of curcumin to reduce oxidative stress and inflammatory response which contributed to insulin resistance. Similarly, curcumin treatment attenuated glucose intolerance and boost insulin sensitizing response [50, 51].

In the present study, the HFD group showed significantly decreased hepatic GSH concentrations and SOD activities in the HFD group when compared with the CD group. These findings were supported by a previous research study that suggested that tissue antioxidant defenses in the HFD group may be reduced [39]. Also, our results were in accordance with Shen et al. [52] who concluded that obesity induced by high-a fat diet involved increased oxidative stress and decreased antioxidant capacity. Likewise, the lipid peroxidation induced by obesity displays a substantial decrease in the level of the main antioxidant enzymes like SOD, GPx, CAT, and GSH [53, 54].

The present study revealed a significant increase in the hepatic GSH and SOD activities in the HFDC group when compared with the HFD group. These results are in accordance with previous studies carried out by Panahi et al. [55] and Farzaei et al. [14], who observed an increase in GSH level and SOD, GPx, and CAT activities and a decrease in MDA level with curcumin treatment. Furthermore, curcumin administration caused strong induction of the antioxidant defenses, since SOD, CAT, and GSH-Px activities were significantly increased, reaching values similar to those of the control group [56, 57]. Curcumin's effects may act by either directly scavenging the reactive oxygen metabolites or due to the presence of various antioxidant principles such as flavonoids, steroids, tannins, glycosides, triterpenoids, and polyphenolic compounds [58–60].

In the present study, hepatic MPO of the HFD group showed significantly higher levels in comparison with the CD group. Parallel with these results, several studies reported that MPO was considered an early biomarker of inflammation and an obesity risk factor in obese individuals [61, 62].

In addition, our results were reinforced by Elgazar-Carmon et al. [63] and Wang et al. [64], who explained that neutrophils infiltrate adipose tissues in the early stage of HFD-induced obesity, promote macrophage infiltration, and release various substances, including reactive oxygen species, TNF-*α,* and MPO, all of which have the capacity to induce inflammation.

In the current study, hepatic MPO of the HFDC group showed significantly lower levels as compared with the HFD group. These results were in agreement with those achieved by Fu et al. [50], who found that curcumin attenuated the infiltration of inflammatory cells and the activity of MPO. Also, our results are in line with Franck et al. [65], who demonstrated that curcumin is a reversible inhibitor of MPO by acting as an excellent electron donor in the peroxidase cycle of the enzyme.

During obesity, TLRs are essential as stimulants for metabolic inflammation and insulin resistance. TLRs, which are known to play a role in innate immunity, are also involved in the inflammatory reactions brought on by HFD. At the molecular level, activation of numerous signaling pathways caused by TLR stimulation leads to the release of proinflammatory cytokines, including IL-1*β*, IL-6, and TNF-*α* [66, 67].

In the current study, the HFD group exhibited significantly higher levels of TLR-4, IL-6 and TNF-*α* gene's expressions in adipose tissues (AT) when compared with the CD group. In the same context, the observations of Tramullas et al. [68] who reported that exposure to long-term high-fat diet induces both peripheral and central alterations in TLR-4 expression resulting in an increased level of proinflammatory cytokines. Also, others like Codoñer-Franch et al. and Heijden et al. reported that AT contains various cell types that all contribute to the inflammatory response results in local and systemic production of numerous soluble products as TNF-*α*, and IL-6 during obesity [69, 70].

The anti-inflammatory effects of curcumin form the basis of its potential clinical applications. In the present study, curcumin significantly reduces the inflammation state in HFDC group indicated by lower levels of TLR-4, IL-6, and TNF-*α* genes' expressions in AT in comparison with HFD group. These observations were confirmed by prior research studies [41, 71, 72]. Additionally, the present results were in accordance with Panahi et al. [55], who revealed that curcumin has been shown to block NF-*κ*B translocation to the nucleus and decrease its DNA binding activity, thereby inhibiting the secretion of proinflammatory cytokines like IL-6 and TNF-*α*.

Curcumin may exert its anti-inflammatory effects through several signaling pathways that are associated with inflammation including the TLR-4 pathway via TLR-4 acting directly on receptor stimulation, by its downstream pathway, or by regulating target proteins in the inflammatory signaling TLR-4 pathway [73, 74].

Our data showed significantly increased DNA fragmentation in the liver homogenates of the HFD group compared to the CD group. Thus, our data agreed with the findings of Yuzefovych et al. [75] and Mu et al. [76], who found a positive association between HFD and DNA fragmentation that was reported in epidemiological studies. Also, this finding was in line with Mendes et al.'s findings [77] that HFD feeding dramatically increased cytoplasmic DNA fragmentation in the heart and liver tissues of HFD fed rats. The possible interpretation provided by Setayesh et al. [78] who emphasize that excess body fat causes DNA damage in multiple organs, including brain, liver, colon, and testes. Different molecular mechanisms may cause genetic instability in overweight/obese individuals.

Interestingly, the current investigation showed significantly decreased DNA fragmentation in liver homogenates of the HFDC group compared to the HFD group. This result was in agreement with many different studies that reported that curcumin compounds have been reported to induce antiproliferative and apoptotic effects. Curcumin is an excellent antioxidant agent and has an effective role in the regression of DNA damage [79, 80].

In the present study, the histopathological observations were done in the liver, duodenum, and heart of the HFD group. These findings were in parallel with van der Heijden et al. [70], Mahmoud et al. [74], and Avtanski et al. [81], who confirmed that the rats fed on a high-fat diet showed marked steatosis in a diffused manner all over the hepatocytes associated with focal necrosis and hepatocellular ballooning.

Additionally, the findings supported those of several researchers who found that HFD consumption resulted in decreased intestinal integrity, intestinal permeability, and histological abnormalities [82–84]. By producing much more ROS and proinflammatory cytokines, oxidative stress and inflammation can both affect the structure and function of the intestinal system, as suggested by Lan et al. [83].

Additionally, our results are in agreement with Chung et al. [85] who assessed the histopathology of the heart and showed significant structural alterations including disordered cardiac muscle fibers and deranged cellular structures in the HFD group.

Interestingly, the current study demonstrated that administration of curcumin to the HFD group provided protection against hepatic disorders, duodenal and myocardial histopathological changes as confirmed by these results are in line with those of many researchers who found a marked reduction in steatosis of hepatocytes and an improvement in liver histopathology [84, 86]. It is likely to postulate that the antioxidative property of curcumin is the key to its therapeutic effect on gastrointestinal injury as documented by many researchers [87–89]. Moreover, Gorabi et al. [90] revealed that high-fat diet plus curcumin showed no histopathological changes in the myocardial structure, indicating a preventive effect of curcumin against histological cardiac changes induced by a high-fat diet.

In general, the current work demonstrated the capacity of our experimental rat model to cause obesity's associated metabolic derangements as shown by biochemical, immunological, and histological alterations brought on by consuming a high-fat diet, which were associated with several microscopic changes in the liver, kidneys, and heart. There is no doubt that the use of curcumin improved the histological findings as well as modulated metabolic and oxidative stress markers, as well as downregulated inflammatory biomarkers and apoptosis. Therefore, it is advised that curcumin be evaluated as a possible supplemental therapy for metabolic diseases linked to obesity brought on by HFD.

## Figures and Tables

**Table 1:** Primer sequences of target genes used for qRT-PCR in this experiment.

GAPDH	Forward	5′-AGACAGCCGCATCTTCTTGT-3′
	Reverse	5′-CTTGCCGTGGGTAGAGTCAT-3′

TLR4	Forward	5-GGGGCAACCGCTGGGAGAGA-3′
	Reverse	5′-AACCAGCGGAGGCCGTGAGA-3′

IL-6	Forward	5′-TCTCTCCGCAAGAGACTTCCA-3′
	Reverse	5′-ATACTGGTCTGTTGTGGGTGG-3

TNF-*α*	Forward	5-ACCACGCTCTTCTGTCTACTG-3′
	Reverse	5′-CTTGGTGGTTTGCTACGAC-3′

## Data Availability

The data used to support the findings of this study are available from the corresponding author upon request.
